# Evidence‐based healthcare competence of social‐ and healthcare educators: A cross‐sectional study

**DOI:** 10.1111/jan.16230

**Published:** 2024-05-10

**Authors:** Kati Immonen, Anna‐Maria Tuomikoski, Kristina Mikkonen, Anne Oikarinen, Saija Ylimäki, Heidi Parisod, Outi Mattila, Maria Kääriäinen

**Affiliations:** ^1^ Research Unit of Health Science and Technology, Faculty of Medicine University of Oulu Oulu Finland; ^2^ The Wellbeing Services County of North Ostrobothnia MRC Oulu Oulu Finland; ^3^ Nursing Research Foundation Helsinki Finland; ^4^ The Finnish Centre for Evidence‐Based Health Care: A JBI Centre of Excellence Helsinki Finland; ^5^ Department of Nursing Science University of Turku Turku Finland; ^6^ Lapland University of Applied Sciences Rovaniemi Finland

**Keywords:** competence, cross‐sectional study, education, educator, evidence‐based healthcare, EBHC, healthcare, social services

## Abstract

**Aim:**

The purpose of the study was to describe social and healthcare educators' evidence‐based healthcare competence and explore the associated factors.

**Design:**

A descriptive, cross‐sectional study was carried out.

**Methods:**

The research spanned 5 universities, 19 universities of applied sciences, and 10 vocational colleges in Finland from September to December 2022. Social and healthcare educators (*n* = 256), of which 21 worked at universities, 176 worked at universities of applied sciences, and 49 worked at vocational colleges. Data collection employed a self‐assessed instrument that was designed to measure evidence‐based healthcare competence based on the JBI Model of Evidence‐based Healthcare. Competence profiles were formed using K‐cluster grouping analysis.

**Results:**

The educators' self‐evaluations of their level of evidence‐based healthcare competence were generally at a satisfactory level, with subsequent analyses identifying four distinct profiles of evidence‐based healthcare competence. The profiles demonstrated statistically significant differences in terms of evidence synthesis and evidence transfer competencies. The factors associated with evidence‐based healthcare competence included level of education, the year in which a professional had obtained their highest degree, current organization of employment, and participation in continuing education.

**Conclusions:**

Educators require various types of support for developing high levels of evidence‐based healthcare competence. The identification of distinct competence profiles can be pivotal to providing educators with training that is tailored to their exact needs to provide an individualized learning path.

**What Problem Did the Study Address?:**

- Educators value the role of evidence in teaching, which reinforces the need to integrate aspects of the JBI Model of evidence‐based healthcare into educators' competencies.
- Aspects of the JBI Model of evidence‐based healthcare have not been holistically measured, with only certain components of the model considered separately.
- Educators need to better understand the global healthcare environment so they can identify research gaps and subsequently develop healthcare systems through their educational role.
- Higher academic education, work experience, organizational support, and continuous education play essential roles in the development of educators' evidence‐based healthcare competence.

**What Were the Main Findings?:**

- Educators generally have high levels of competence in evidence‐based healthcare.
- Educators have mastered the different components of the JBI model of evidence‐based healthcare but need to improve in areas such as the transfer and implementation of evidence.

**Where and on Whom Will the Research Have an Impact?:**

- Determining evidence‐based healthcare competence profiles for educators can be used to provide individualized learning paths for the development of evidence‐based healthcare competence.
- Educators need to further develop their competence in evidence‐based healthcare to ensure successful implementation and high‐quality education in the future.

**Patient or Public Contribution:**

No patient or public contribution.

## 1 INTRODUCTION

Social and healthcare professionals must be competent in evidence‐based healthcare (EBHC) to ensure high‐quality, safe, effective, and meaningful care to patients; this has become increasingly noticeable in the constantly changing and evolving context of social and healthcare (Jordan et al., 2019; Lockwood, 2017; Melnyk et al., 2018; Mikkonen et al., 2019; WHO, 2016). Educators are at the forefront of translating EBHC theory into the clinical setting, as they are responsible for providing high‐quality education to future health professionals (Immonen et al., 2022; Kuivila et al., 2020).

A social and healthcare educator's proficiency in teaching is linked to the competencies they need to design and implement education that reflects the current field. Various studies have defined distinct areas of an educator's competence (Gibson et al., 2019; Leung et al., 2016; Mikkonen et al., 2018, 2019; Moynihan et al., 2015; WHO, 2016), which is generally considered a multidimensional concept. As such, an educator's proficiency includes both ‘micro‐level’ (pedagogy, ethics, culture, interaction, collaboration and networking, administration and welfare, EBHC) and ‘macro‐level’ competencies (sustainable innovation and continuing competence development) (Mikkonen et al., 2019). Educators who strive to have sufficient EBHC competence must continuously consider the best evidence available in decision‐making when educating students, collaboration with colleagues, and the development of new educational methods (Jordan et al., 2019; Mikkonen et al., 2018).

## 2 BACKGROUND

The JBI model of EBHC, which describes the steps needed to implement evidence in healthcare, considers education to have a crucial role in this process (Jordan et al., 2019; Pearson et al., 2005). The model comprises five components: global health, evidence generation, evidence synthesis, evidence transfer and evidence implementation (Jordan et al., 2019). As such, educators must have strong competence in each of these five areas if they hope to sufficiently educate students in evidence implementation. A part of the global health aspect of the JBI model of EBHC requires educators to be able to identify knowledge needs related to global health in close collaboration with students, health professionals, patients/clients, governments and other organizations (Immonen et al., 2022). For example, educators may mentor students in clinical placements, a relationship during which students participate in the development of evidence‐based nursing care and respond to patients' needs (Mikkonen, Tomietto, Tuomikoski, et al., 2022).

Evidence generation is defined as the formal means of producing knowledge through discourse, experience and research – a competence which requires educators to understand the research process (Jordan et al., 2019), from defining concepts to analysing research data (Halvari et al., 2021). Evidence synthesis is concerned with how a professional evaluates, analyses and/or synthesizes research evidence to support healthcare decision‐making (Jordan et al., 2019). It is important for educators to understand the process of evidence synthesis and possibly contribute to implementation by leveraging their own competencies. A social and healthcare education should also understand how to use synthesized evidence (e.g. systematic reviews, evidence summaries and guidelines) as part of the teaching process (Immonen et al., 2022).

Evidence transfer describes the set of factors that enable, facilitate and support evidence implementation. As such, this aspect of EBHC includes active evidence dissemination, education and clinical integration (Jylhä et al., 2017; Jordan et al., 2019; Munn et al., 2018). For example, educators must be able to actively disseminate evidence and evidence‐based practices through teaching and mentoring, both in different working environments and on national and international levels (Immonen et al., 2022). Evidence implementation – in the context of the JBI Model of EBHC – involves a purposeful set of activities designed to provide stakeholders with the evidence necessary to inform decision‐making and generate high‐quality care delivery (Jordan et al., 2019). This area of the JBI model of EBHC specifies that the educator has a role in clarifying the principles underlying the development evidence‐based practice, as well as monitoring teaching and mentoring (Mikkonen et al., 2019). However, social and healthcare educators who wish to implement evidence in clinical care need to collaborate with other professionals and discuss with managers (Immonen et al., 2022; Li et al., 2018).

The requirements for acting as an academic social and healthcare educator differ among European countries (European Commission, 2020). A general definition for a social and healthcare educator is a professional holding a master's degree in health sciences and pedagogical accreditation defined by the educator's teaching experiences and/or education (Government Decree of the Universities of Applied Sciences 1129/2014). For example, in Finland, universities are responsible for providing the pedagogical education that social and healthcare educators require (Government Decree on Universities 770/2009). The social and healthcare educators working at universities of applied sciences educate professionals such as registered nurses and midwives (Government Decree on Polytechnics 1129/2014), while the educators at vocational colleges educate students who wish to work as practical nurses at old peoples' homes and day care centres (Government Decree on Vocational colleges 673/2017).

Previously published literature concerning social and healthcare educators reveals that the five aspects of the JBI model of EBHC have not yet been comprehensively studied. Educators' competence in evidence‐based practice has been measured with various instruments (Lemetti et al., 2023; Mikkonen et al., 2018, 2020; Nielsen et al., 2024; Salminen et al., 2021), but no study – to the best of our knowledge – has included specific measurements of EBHC competence. In addition, EBHC competence comprises various skills related to teaching rather than the ability to understand, evaluate and apply evidence in decision‐making. There is also a lack of knowledge about the distribution of educators' EBHC competence, or which components of EBHC competence should be highlighted in continuing education for educators. Previous research has reported that academic education, work experience, continuous education and organizational support are all positively associated with an educator's competence in different areas of EBHC (Erkkilä et al., 2023; Immonen et al., 2022; Koivula et al., 2011). However, prior studies have not explored the competence in all areas of EBHC.

## 3 THE STUDY

### 3.1 Aim

The aim of this study was to describe social and healthcare educators' EBHC competence and explore associated factors. The research was guided by the following research questions: (1) What EBHC competence levels are present among social and healthcare educators in higher education and vocational colleges, and are there distinct EBHC competence profiles?; and (2) What are differences between EBHC competence profiles in terms of demographic characteristics?

## 4 METHODS

### 4.1 Design

This study used a descriptive cross‐sectional study design to describe the variables and analyse their occurrence and interrelationship at a given point in time.

### 4.2 Participants and setting

A total of 2106 educators from all five Finnish‐speaking universities in the field of health sciences, all 19 universities of applied sciences in Finland, and 10 randomly selected vocational colleges were invited to participate in the study. The inclusion criteria for participation were working as a full‐time or part‐time social and healthcare educator; and an employee of a university, university of applied sciences, or vocational school at the time of the study. No inclusion criteria for pedagogical education were set. A total of 256 (12.2% of the target group) educators participated in the study.

### 4.3 Instrument

The self‐assessed Evidence‐Based Healthcare Competence of Educators (EBHC‐COMP‐Edu) instrument was used to measure EBHC competence. The instrument was developed at the University of Oulu, in collaboration with the Nursing Research Foundation, based on the JBI Model of EBHC (Jordan et al., 2019) and several systematic reviews (Härkönen et al., 2021; Immonen et al., 2022; Kanste et al., 2021; Koivunen et al., 2023; Ylimäki et al., 2024) that focused on the general EBHC competence needs of healthcare professionals, advanced practice nurses, educators and leaders. In this study, an additional sub‐dimension of EBHC competence, which was identified as being pertinent to educators in a recent systematic review (Immonen et al., 2022), was added to the instrument.

The instrument included 37 items and six sub‐dimensions, namely, knowledge needs related to global health (12 items), Evidence Generation (nine items), Evidence Synthesis (four items), Evidence Transfer (three items) and Evidence Implementation (three items), along with the additional sub‐dimension of teaching and supervision of evidence‐based healthcare (six items). Items were formulated so that they could be assessed using a five‐point Likert rating scale (1 – very poor, 2 – poor, 3 – moderate, 4 – satisfactory, 5 – excellent competence). In addition, the instrument included 14 background questions related to educators' gender, age, previous education, year of completion of highest educational qualification, current field of education, current pedagogical education, continuing education, area of current work and job description, current work organization, participation in research projects, and participation in development projects.

In this study, the content validity of the instrument was evaluated using Content Validity Index (CVI), which provides information about validity of items included in the instrument (Kääriäinen et al., 2020). The EBHC experts (*n* = 8) (nursing educators, nursing leaders, researchers) rated independently the suitability and relevance of the items using online 4‐point scale (from 1 = not relevant to 4 = very relevant) which was accessible via online Webropol survey (Webropol, Helsinki, Finland). Based on responses both the individual item CVI (I‐CVI) and the total average score (S‐CVI/Ave) were calculated (Polit et al., 2007). The threshold for an acceptable I‐CVI score was set at ≥0.70, while the acceptable S‐CVI‐Ave range was set as 0.70–1.00 (Polit et al., 2007). In this study, the I‐CVI values ranged between 0.62 and 1, while the S‐CVI/Ave was 0.96. Items that received I‐CVI scores lower than 0.70 were modified according to the comments provided by experts and later re‐evaluated by a small group of researchers. After ensuring content validity, the instrument was pre‐tested on two healthcare professionals. These two participants were asked to assess the understandability, clarity and duration of the instrument.

The results of Bartlett's sphere test (*p* < .001) and the Kaiser–Meyer–Olkin test (0.953) demonstrated that the sample of professionals (*n* = 256) was adequate for testing the construct validity of the instrument. First, principal component analysis (PCA) was used to identify correlated items and cross‐loading; next, the PCA results were verified using exploratory factor analysis (EFA). The EFA was conducted using Principal Axis Factoring and Promax rotation (see Table 1). The construct validity of the instrument was tested using data from our sample of social and healthcare educators (*n* = 256). During EFA, none of the original items was removed because communalities were between 0.33 and 0.92 and all of the factor loadings were >0.30 (Yong & Pearce, 2013). The suggested cut‐off communality values for this type of analysis fall between 0.25 and 0.4, with ideal values exceeding 0.7, so this was an appropriate result (Beavers et al., 2013). The conducted EFA yielded a six‐factor structure comprising 37 items.

**TABLE 1:** Exploratory factor analysis of the EBHC Competence Instrument.

Factor describing a social‐, healthcare and rehabilitation educators evidence‐based healthcare competence	Items including in the factor. *1 = completely disagree, 2 = partially disagree, 3 = partially agree, 4 = completely agree*	Factor 1	Factor 2	Factor 3	Factor 4	Factor 5	Factor 6
Knowledge needs related to the global health	I can guide student to assess the usefulness of service/care or rehabilitation methods in different situations	0.926					
	I can guide student to assess the appropriateness of service/care or rehabilitation methods in different situations	0.906					
	I can guide students to critically evaluate their own activities	0.783					
	I can guide students to critically evaluate the practices of the work community	0.771					
	I can guide the student to present information needs related to service/care/rehabilitation development	0.712					
	I can guide students to evaluate the effectiveness of service/treatment or rehabilitation methods	0.626					
	I can guide the student to take into account the patient's/client's wishes (relevance) in care	0.609					
	I can support students to engage in evidence‐based practice	0.467					
Evidence generation	I can evaluate different sources of information (research, expertise, experience)	0.844					
	I can apply different research methods		0.792				
	I can critically assess the main factors related to the reliability of research		0.763				
	I can apply the different steps of the research process in my work		0.756				
	I can apply theory‐based research ethics principles in my work		0.688				
	I can independently search for research information in the most common databases (e.g. PubMed, CINAHL, Medline)	0.491					
	I can critically evaluate the applicability of evidence in teaching (e.g. the suitability of the use of treatment recommendations, systematic reviews as part of teaching)	0.394					
Evidence transfer	I can transfer evidence through my national networks			0.788			
	I can be an active disseminator of evidence in my work, e.g. in meetings, trainings, mentoring, consulting (e.g. care recommendations, reviews)	0.730					
	I can develop the curriculum taking into account the objectives of evidence‐based social, health and rehabilitation services	0.665					
	I can develop my own social, health and rehabilitation evidence‐based competence skills	0.642					
	I can strengthen the evidence‐based healthcare skills of future social, health and rehabilitation professionals	0.637					
	I can work with a service organization to promote evidence‐based social, health and rehabilitation services	0.615					
	I can transfer evidence through my international networks			0.576			
	I can guide/teach students to choose the appropriate evidence‐based service/treatment or rehabilitation method for the client/patient in accordance with the organization's standard practices	0.562					
	I can guide students to seek systematic reviews and treatment recommendations to support the development of their own expertise, activities and decision‐making.	0.436					
Evidence synthesis	I can explain the steps in the process of developing a treatment recommendation			0.839			
	I can explain the steps involved in writing a screen summary (e.g. a screen tip)			0.729			
	I can explain the importance of the evidence level in developing practices				0.429		
	I can use, e.g. PICO, PICo or PCC tools or other similar data/research evidence for teaching purposes	0.306	0.405				
	I can carry out a systematic review as part of a team				0.403		
Teaching and Supervision of Evidence‐based Healthcare	I can explain the concept of an evidence					0.720	
	I can explain the objectives of evidence‐based practice					0.657	
	I can explain the process of evidence‐based practice (evidence generation, implementation and verification)	0.354	0.633				
	I can explain the steps of the evidence‐based healthcare (JBI) model (identifying the need for information, generating research evidence, summarizing the evidence into evidence, disseminating the evidence and implementing the evidence)	0.465	0.586				
	I can explain the importance of the level of evidence in clinical decision‐making			0.388	0.391		
Evidence implementation	I can guide students to act in accordance with evidence‐based consistent practices		0.331			0.726	
	I can guide students to make decisions based on evidence or expert knowledge					0.560	
	I can guide learners to assess the progress of evidence‐based integrated practices			0.341		0.500	
Eigenvalue		18.39	2.98	1.61	1.33	1.08	0.91
Percentage of variance (%)		49.7	8.06	4.35	3.61	2.93	2.46
Total percentage of factor model (%)							71.15
Cronbach's alpha for each factor		0.917	0.912	0.928	0.89	0.89	0.89
Cronbach's alpha on total scale							0.971
Extraction method: Principal Axis Factoring. Rotation method: Promax with Kaiser Normalization							

The first factor, knowledge needs related to global health, had an eigenvalue of 18.39. This factor included eight items and explained 49.70% of the total variance. The second factor, Evidence generation, had an eigenvalue of 2.98, consisted of seven items, and explained 8.06% of the total variance. The third factor, Evidence transfer, had an eigenvalue of 1.61, consisted of nine items, and explained 4.35% of the total variance. The fourth factor, Evidence synthesis, had an eigenvalue of 1.33, consisted of five items and explained 3.61% of the total variance. The fifth factor, teaching and supervision of evidence‐based healthcare, had an eigenvalue of 1.08, consisted of five items and explained 2.93% of the total variance. The sixth, and final factor (Evidence implementation) had an eigenvalue of 0.91, included three items and explained 2.46% of the total variance. The acceptable range of explained variance is generally at least 50%–60% (Pett et al., 2003), which is in line with our results, as the six aforementioned factors explained 71.15% of the total variance (Table 1).

The internal consistency of the 37‐item instrument was evaluated by calculating Cronbach's alpha, which ranged from 0.89 to 0.93 for the six factors; more specifically, *α* = 0.92 for knowledge needs related to global health (eight items), *α* = 0.91 for Evidence Generation (seven items), *α* = 0.93 for Evidence Transfer (nine items), *α* = 0.89 for Evidence Synthesis (five items), *α* = 0.89 for teaching and supervision of evidence‐based healthcare (five items) and *α* = 0.89 for Evidence Implementation (three items). The instrument demonstrated sufficient internal consistency (DeVon et al., 2007).

### 4.4 Data collection

The data were collected using an electronic questionnaire from September to December 2022. The contact person at each participating educational organization sent a link to the study by email to educators who met the inclusion criteria. The invitation was sent once, with four reminder messages sent every month. The survey was conducted in Finnish, with the results back‐and‐forth translated into English.

### 4.5 Data analysis

Descriptive statistics (frequency, percentage, mean, median and standard deviation) were obtained through IBM SPSS Statistics (version 25; IBM, Armonk, NY). The scores for the items included across the six factors of the instrument were summated, with this information input into a k‐means cluster analysis, which was used to identify competence profiles. Cluster analysis is used to identify sub‐groups of the data used, whose members are similar in certain characteristics and at the same time differ from members of other groups. After running four different models, four profiles (A, B, C, D) were identified, and found to be the most suitable in terms of the aim of this study. The following score ranges described different levels of EBHC competence: <2.49 – low level of competence, 2.50–3.49 – moderate level of competence, 3.50–4.49 – satisfactory level of competence and >4.50 – excellent level of competence.

At first, three different cluster models were applied, which yielded five, four and three clusters of competence, respectively, with the criterion that no cluster could involve less than 5% of the total sample. After these preliminary results, a fourth cluster model was chosen and demonstrated the best performance. The significance of differences between the educators' competence profiles was determined using a Kruskal–Walli's test, with the results further analysed using a Bonferroni post‐hoc test, which was performed by conducting a Mann–Whitney test. The significance of differences in background characteristics was analysed using a Chi‐squared test and one‐way analysis of variance (ANOVA). A two‐tailed *p*‐value <.05 was considered to be statistically significant.

### 4.6 Ethical considerations

All stages of the research were carried out in accordance with good scientific practice (RCR, 2012). Appropriate research permits were gathered from all of the organizations included in the study in 2022. The Research Ethics Committee statement was not required according to guidelines from the Finnish National Board on Research Integrity (TENK, 2023) since the study did not influence the physical and/or mental integrity of participants, involve underaged children or use non‐consent registered data (Declaration of Helsinki, 2013). All of the research data were stored and processed in line with data protection principles and legislation (Data Protection Act 1050/2018; GDPR, 2016). Participation in the survey was voluntary and informative. Each participant received information about the study in a letter, which covered the purpose of the study, the participant's rights the researchers' contact details and a link to the questionnaire. Each participant was required to provide informed consent before progressing to the survey questions.

## 5 RESULTS

### 5.1 Sample characteristics

The respondents were between 27 and 68 years of age (mean 49.4, SD 8.78), and most of the participants (87.5%) were female. Most of the participants worked in healthcare education (71%), with others working in rehabilitation education (14%) and social work education (11%). Concerning education, 19.1% of the participants held a Doctoral degree (PhD), while 77% had a Master's university degree or a higher vocational degree; the remainder of the participants (4%) had a Bachelor's university degree or vocational diploma.

Most of the participants worked at universities of applied sciences (69%), with the rest representing vocational colleges (23%) and universities (*n* = 8%). In terms of pedagogical education, 52% of the participants had completed a teacher education in health sciences, while 35% had completed a vocational teacher education and 9% had completed a teacher education in educational science. Only 4% of the participants had not completed any form of teacher education. A majority of the participants were working as lecturers at a university of applied sciences (51%), with the remainder working as full‐time educators (15%), vocational education educators (14%), principal lecturers (9%), head of education or doctoral researchers (7%), university lecturers (4%) and professors (1%). Over the last 2 years, 52% of participants had participated in at least 1–4 development or research projects, while 48% had participated in more than four courses (Table 2).

**TABLE 2:** Characteristic of participants.

Characteristic (*n* = 256)		*n*	*%*
Gender	Male	28	11
	Female	224	87.5
	Not want to tell	4	1.5
Age^a^	<35 years	17	6.6
	35–40 years	29	11.3
	41–50 years	90	35.2
	51–60 years	93	36.3
	>60 years	27	10.5
Highest education level	Vocational degree	3	1.2
	University (Bachelor's) degree	8	3.1
	University (Master's) degree	196	76.6
	University (Doctoral) degree	49	19.1
Educator training (pedagogical education)	Vocational teacher education	90	35.2
	Teacher education of health sciences	132	51.6
	Teacher education in educational sciences	24	9.4
	No teacher education	10	3.9
Current teacher work field	Social services	29	11.3
	Healthcare	182	71.1
	Rehabilitation	36	14.1
	Education in sports	9	3.5
Current work organization	Vocational school	59	23
	University of Applied Sciences	176	68.8
	University	21	8.2
Current employment	Full‐time educator	37	14.5
	Vocational school educator	36	14.1
	University of applied sciences educator	130	50.8
	University educator	9	3.5
	Principal educator	24	9.4
	Professor	3	1.2
	Head of Training, doctoral researcher, etc.	17	6.6
Participation in development or research projects	At least in 1–4 development or research projects	132	51.6
	At least in 5–10 development or research projects	124	48.4

^a^ Mean value: 49.4 years (SD = 8.78, range 27–68 years).

### 5.2 Overall EBHC competence level among the social and healthcare educators

The overall mean value of educators' EBHC competence varied from moderate to satisfactory across the six sub‐categories: knowledge needs related to global health mean (3.93), evidence generation (3.95), evidence transfer (mean 3.53), evidence synthesis (mean 3.15), teaching and supervising evidence‐based healthcare (SD 3.77), and evidence implementation (SD 3.73) (see Table 3).

**TABLE 3:** Demographic characteristics of educators (*n* = 256).

Characteristics and competence	Profile A (*n = 34*)	Profile B (*n = 87*)	Profile C (*n = 84*)	Profile D (*n = 51*)		*p*‐value
Age in years, mean (SD)^a^	50.50 (8.41)	49.08 (8.61)	48.88 (10.56)	49.37 (8.98)		.856
Gender %						.216
Female	85.3 (*n* = 29)	89.7 (*n* = 78)	84.5 (*n* = 71)	90.2 (*n* = 46)		
Male	8.8 (*n* = 3)	8 (*n* = 7)	15.5 (*n* = 13)	9.8 (*n* = 5)		
Not want to tell	5.9 (*n* = 2)	2.3 (*n* = 2)	0 (*n* = 0)	0 (*n* = 0)		
Education						**<.001**
Vocational degree	5.9 (*n* = 2)	1.2 (*n* = 1)	0 (*n* = 0)	0 (*n* = 0)		
University (Bachelor's) degree	0 (*n* = 0)	4.7 (*n* = 4)	2.4 (*n* = 1)	3.9 (*n* = 2)		
University (Master's) degree	91.2 (*n* = 24)	87.2 (*n* = 59)	75.9 (*n* = 54)	49 (*n* = 21)		
University (Doctoral) degree	2.9 (*n* = 1)	7 (*n* = 6)	21.7 (*n* = 18)	47.1 (*n* = 24)		
The year of completion of the highest degree (mean) SD	2007 (8.53)	2012 (6.54)	2012 (7.08)	2011 (7.43)		.008
Educator training (pedagogical education), %						.057
Vocational teacher education	44.1 (*n* = 15)	40.2 (*n* = 35)	29.8 (*n* = 25)	29.4 (*n* = 15)		
Teacher education of health sciences	32.4 (*n* = 11)	46.0 (*n* = 40)	57.1 (*n* = 48)	64.7 (*n* = 33)		
Teacher education in educational sciences	20.6 (*n* = 7)	10.3 (*n* = 9)	8.3 (*n* = 7)	2 (*n* = 1)		
No teacher education	2.9 (*n* = 1)	3.4 (*n* = 3)	4.8 (*n* = 4)	3.9 (*n* = 2)		
Current educator work field, %						.181
Social services	23.5 (*n* = 8)	13.8 (*n* = 12)	9.5 (*n* = 8)	2 (*n* = 1)		
Healthcare	61.8 (*n* = 21)	72.4 (*n* = 63)	69.0 (*n* = 58)	78.4 (*n* = 40)		
Rehabilitation	11.8 (*n* = 4)	11.5 (*n* = 10)	17.9 (*n* = 15)	13.7 (*n* = 7)		
Education in sports	2.9 (*n* = 1)	2.3 (*n* = 2)	3.6 (*n* = 3)	5.9 (*n* = 3)		
Current employment, %						.005
Part‐time educator	0 (*n* = 0)	2.3 (*n* = 2)	2.4 (*n* = 2)	0 (*n* = 0)		
Full‐time educator	8.8 (*n* = 3)	16.1 (*n* = 14)	14.3 (*n* = 12)	7.8 (*n* = 4)		
Vocational school educator	29.4 (*n* = 10)	19.5 (*n* = 17)	8.3 (*n* = 7)	3.9 (*n* = 2)		
University of applied sciences educator	52.9 (*n* = 18)	50.6 (*n* = 44)	51.2 (*n* = 43)	49.0 (*n* = 25)		
University educator	0 (*n* = 0)	0 (*n* = 0)	3.6 (*n* = 3)	11.8 (*n* = 6)		
Principal educator	2.9 (*n* = 1)	5.7 (*n* = 5)	10.7 (*n* = 9)	17.6 (*n* = 9)		
Professor	0 (*n* = 0)	0 (*n* = 0)	2.4 (*n* = 2)	2 (*n* = 1)		
Head of Training, doctoral researcher, etc.	5.9 (*n* = 2)	5.7 (*n* = 5)	7.1 (*n* = 6)	7.8 (*n* = 4)		
Current work organization, %						**<.001**
Vocational school	35.5 (*n* = 11)	24.2 (*n* = 20)	18.1 (*n* = 15)	6.0 (*n* = 3)		
University of Applied Sciences	61.3 (*n* = 19)	74.4 (*n* = 61)	68.7 (*n* = 57)	78.0 (*n* = 39)		
University	3.2 (*n* = 1)	1.2 (*n* = 1)	13.3 (*n* = 11)	16.0 (*n* = 8)		
Continuing education						
Participated in development projects	70.6 (*n* = 24)	77.9 (*n* = 67)	90.5 (*n* = 76)	92.2 (*n* = 47)		**<.007**
Participated in research projects	47.1 (*n* = 16)	46 (*n* = 40)	69.0 (*n* = 58)	78.4 (*n* = 40)		**<.001**
Participated in less than four training courses in 2 years	70.6 (*n* = 24)	63.2 (*n* = 55)	42.9 (*n* = 36)	33.3 (*n* = 17)		**<.001**
Participated in more than four training courses in 2 years	29.4 (*n* = 10)	36.8 (*n* = 32)	57.1 (*n* = 48)	66.7 (*n* = 34)		**<.001**
					**Average of overall competencies**	
Knowledge needs related to The Global Health	3.31 (0.59)	3.67 (0.41)	4.06 (0.51)	4.56 (0.35)	3.93	**<.001** ^b^
Evidence generation	3.05 (0.42)	3.65 (0.39)	4.13 (0.36)	4.74 (0.33)	3.95	**<.001** ^c^
Evidence transfer	2.37 (0.42)	3.20 (0.33)	3.75 (0.34)	4.50 (0.38)	3.53	**<.001** ^b^
Evidence synthesis	1.85 (0.46)	2.74 (0.47)	3.33 (0.48)	4.45 (0.45)	3.15	**<.001** ^b^
Teaching and Supervision of evidence‐based healthcare	2.65 (0.51)	3.38 (0.42)	4.01 (0.46)	4.78 (0.31)	3.77	**<.001** ^c^
Evidence implementation	2.61 (0.62)	3.37 (0.43)	4.11 (0.40)	4.49 (0.53)	3.73	**<.001** ^c^
Educators' overall EBHC competence	mean 2.64	mean 3.33	mean 3.89	mean 4.58		

*Note*: *p* < .05 (marked in bold).

^a^ M: mean (SD: standard deviation).

^b^ One‐way ANOVA test.

^c^ Kruskal–Walli's test, Mann–Whitney test used for Bonferroni correction.

### 5.3 Educators' EBHC competence profiles

Profile A included 13% (*n* = 34) of the participants. Educators in this profile had an average age of 50 years and were most likely to have completed their highest degree in 2007. More than 90% of Profile A educators held a master's degree. The pedagogical education level of the educators was evenly distributed, that is, vocational teacher education (44%), teacher education in health science (32%) and teacher education in educational sciences (21%). Most Profile A educators worked in healthcare (62%), while this profile also demonstrated the largest representation of professionals working in social services (24%). Profile A educators worked at either a university of applied sciences (61%) or in vocational education (36%). Moreover, most Profile A educators had participated in development projects (71%), with 47% having experience in research projects during the last 2 years. In terms of continuous education, 71% of Profile A educators had participated in less than four education courses in the last 2 years, while 29.4% had participated in more than four education courses in the last 2 years.

The mean overall EBHC competence of educators ranged from a minimum of 2.64 to a maximum of 4.58 across the four identified competence profiles. Profile A educators gave the highest score to their competence in knowledge needs related to global health the highest (mean 3.3, SD 0.59) and the lowest score to competence in evidence synthesis (mean 1.8, SD 0.46). Profile A educators evaluated their competence in evidence generation (mean 3.0, SD 0.42) and teaching and supervision of evidence‐based healthcare (mean 2.6, SD 0.51) as moderate, while the self‐assessments of competence in evidence implementation (mean 2.61, SD 0.62) and evidence transfer (mean 2.4, SD 0.42) were at a poor level.

Profile B included 34% (*n* = 87) of the participants. Educators in this profile had an average age of 49 years and were most likely to have completed their highest degree in 2012. A clear majority of the educators who belonged to Profile B held a Master's degree (87%), followed by a doctoral degree (7%). In terms of pedagogical education level, the most common qualification was teacher education in health science (46%), followed by vocational teacher education (40%) and teacher education in educational sciences (10%). Most of the Profile B educators worked in healthcare (72%), with the remainder working in social services (14%), rehabilitation sector (12%) and education in sports (2%). Half of Profile B educators worked at a university of applied sciences, with 20% involved in vocational education and 16% working as full‐time educators. A large share of Profile B educators had participated in development projects (78%), with 46% participating in research projects during the last 2 years. In terms of continuous education, over half of the educators (63%) had participated in less than four education courses over the last 2 years, while 37% of Profile B educators had participated in more than four education courses over the last 2 years. Profile B educators' self‐assessments yielded mean values of competence that fell under either moderate or satisfactory levels across all of the sub‐dimensions of EBHC competence. Profile B educators scored their competence in knowledge needs related to global health and evidence generation the highest (mean 3.6, SD 0.41) and their competence in evidence synthesis the lowest (mean 2.7, SD 0.47).

Profile C included 33% (*n* = 84) of the participants, with an average age of 49 years and 2012 as the average year in which educators had received their highest degree. Educators in Profile C mostly had a Master's degree (76%), while 22% had a doctoral degree. In terms of pedagogical education level, more than half (57%) of the educators had completed teacher education in health science, followed by vocational teacher education (30%). The majority of Profile C educators worked in healthcare (69%), followed by rehabilitation (18%). Profile C educators were most likely to work in a university of applied sciences (51%), while 15% worked as full‐time educators and 11% worked as principal educators. Almost all of the Profile C educators had participated in development projects (91%), with a majority (69%) also being part of research projects. In terms of continuous education, 43% of Profile C educators had participated in less than four education courses over the last 2 years, while 57% had participated in more than four education courses over the last 2 years. Profile C educators' self‐assessments of competence revealed mean scores associated with moderate or satisfactory levels across all of the sub‐dimensions of EBHC competence. Profile C educators scored their competence in evidence generation the highest (mean 4.1, SD 0.36) and their competence in evidence synthesis the lowest (mean 3.3, SD 0.48).

Profile D included 20% of the participants. Educators in this profile had an average age of 49 years and were most likely to have received their highest degree in 2011. Profile D had the highest share of educators holding a doctoral degree (47%), while 49% of Profile D educators had a Master's degree. Regarding pedagogical education level, a clear majority of Profile D educators (65%) had completed teacher education in health science, followed by vocational teacher education (30%). A large share (78%) of Profile D educators worked in healthcare. Profile D educators worked at universities of applied sciences (49%), as principal educators (18%), and universities (12%). A majority of Profile D educators had participated in both development (92%) and research (78%) projects. In terms of continuous education, 67% had participated in more than four education courses in the last 2 years, while 33% had participated in less than four education courses over the 2 years. Profile D educators reported satisfactory or excellent levels of competence across all of the sub‐dimensions of EBHC competence. Generally, Profile D educators reported that they were most competent in teaching and supervision of evidence‐based healthcare and evidence generation (mean 4.7, SD 0.31), yet the least competent in evidence synthesis (mean 4.5, SD 0.45).

### 5.4 Differences between EBHC competence profiles in terms of demographic characteristics

All six sub‐dimensions of EBHC competence showed statistically significant differences between the four educator profiles (*p* < .001). The profiles were also found to significantly differ in terms of current work organization (*p* < .001). Moreover, statistically significant between profile differences were found in education, current employment and participation in continuing education (*p* < .001). For instance, the average year in which an educator received their highest educational degree was 2007 for Profile A, which was significantly earlier than Profiles B–D (between 2011 and 2012). Educators belonging to Profiles C and D were significantly more likely to have participated (more than five times over 2 years) in educational courses than educators representing Profiles A and B. A significantly larger share of Profile C and Profile D educators had participated in development projects (over 90%) relative to educators in Profiles A and B; this also held true for the case of research projects, with educators in Profiles C (69%) and D (78%) showing significantly higher participation rates than educators representing Profiles A and B (Table 3).

## 6 DISCUSSION

This study aimed to describe social and healthcare educators' EBHC competence and explore associated factors. Although educators' competencies have been identified as good level in the past (Halvari et al., 2021; Immonen et al., 2022), competencies assessed according to the JBI EBHC model clearly highlighted gaps in certain sub‐areas.

The presented results revealed that education level influences an educator's self‐assessed EBHC competence. Educators belonging to Profile D were the most competent in all sub‐dimensions of EBHC competence. This profile had the highest number of educators from universities of applied sciences and universities, as well as the highest representation by participants with a doctoral degree (47%). It is logical that doctoral graduates, who boast the strongest experience in EBHC theory, would have the strongest competence. Profile C also included professionals with a doctoral degree (22%), as well as strong representation by participants with a master's degree (76%). In comparison, Profile A had the lowest share of educators with a PhD, and the highest share of educators working at vocational education colleges. The results thus indicate that an educator's educational level may strengthen their EBHC competence. This may be explained by educational organizations placing different emphases on various aspects of EBHC in the corresponding teaching, along with different requirements of the extent to which teachers must master EBHC. It is important to note that previous research has also reported that educational level impacts educators' competence in EBHC (Immonen et al., 2022; Koivula et al., 2011).

The educational programmes provided at universities and universities of applied sciences generally include a strong emphasis on research methods and evidence‐based practices; this characteristic can partly explain the stronger levels of EBHC competence witnessed among educators working in these types of organizations. It has been established that EBHC is embedded – to some extent – into universities and universities of applied sciences (Halvari et al., 2021; Kuivila et al., 2020). However, the differences in EBHC competence levels among Finnish educators demonstrate how EBHC must be better taught across all educational institutions. In addition, educators should be able to actively incorporate the latest evidence into curricula. Although the participating educators were found to have a good command of the sub‐dimensions of EBHC, they must also be competent at explaining the significance of these aspects to students. According to Halvari et al. (2021), it would be important to utilize the competence of educators in the different components of the JBI model of EBHC. In other words, educators must be able to use, apply and integrate these competencies in their daily practice in both academic and clinical settings.

The presented results demonstrate that evidence‐based practices are clearly considered a part of social and healthcare curricula, with most participants understanding that the use of research material is important for teaching and the overall development of education. The results of earlier studies have also shown that educators have a positive attitude towards the implementation of evidence‐based teaching and are therefore ready to find ways to effectively integrate research into their teaching (Diery et al., 2020, 2021; Nielsen et al., 2024). The fact that some of these factors are already embedded in educator education programmes (Mikkonen, Sorvari, Kuivila, et al., 2022) means that there is a strong probability that educators will incorporate the various sub‐dimensions of EBHC in their daily work.

In this study, the average year in which an educator completed their highest academic degree also significantly varied between the different profiles. Educators who belonged to Profile A showed the earliest average time of completing their highest degree (the year 2007), when compared to the years 2011–2012 for Profiles B–D. This significant difference in the time of graduation could impact educators' competence levels because the JBI model of EBHC is recent and may not have been integrated into social and healthcare curricula over a decade ago. The JBI model of EBHC, which provides a framework that guides clinical decision‐making along with the incorporation of scientific evidence from well‐designed studies (Melnyk et al., 2018), although integrated into curricula, is not yet as visible in the teaching. Thus, continuing education is critical for educators who wish to provide students with high‐quality education, and this is particularly relevant for educators who have completed their own degree over 10 years ago. To strengthen EBHC as an integral part of education policy, educators' EBHC competence assessments could be introduced as part of educators' development discussions. This would allow for better support in competence management in organizations to meet educators' individual development needs of EBHC competence.

In this study, EBHC competence challenges emerged in the areas of evidence synthesis and transfer, which can be explained by the fact that the definitions of these concepts are not generally well understood and used to a low extent in education. This is also supported by previous research, which has revealed a lack of holistic management, especially from an educational perspective (Halvari et al., 2021; Immonen et al., 2022). The most significant competence differences between Profiles A and D concerned evidence synthesis competence and evidence transfer competence. The high level of expertise in evidence synthesis among members of Profile D can be, at least partly, connected with the high educational levels found in this profile. This is also supported by previous research, as there are reports that high educational levels are associated with high levels of evidence‐based competence (Koivula et al., 2011). Doctoral studies or working as an educator at a university might strengthen an educator's competence in evidence synthesis, including the understanding of what this process entails. This is because work at a university involves a strong focus on research; this will develop an educator's knowledge and experience with how to incorporate the latest research into their daily work.

Regarding vocational education, mastering the entire JBI model of EBHC may not necessarily seem from the educator's point of view. Within the same perspective, the synthesis or transferring of evidence may not be considered an essential part of daily teaching work. However, all educators, whether they graduated from a university, a university of applied sciences, or a vocational college, should master the basics of the JBI model of EBHC. Prior research has also evaluated the research skills of social and healthcare educators, and revealed satisfactory competence levels, which agrees what was reported in the present study (Immonen et al., 2022; Kuivila et al., 2020).

According to the results presented in this study, educators' participation in research and development projects is associated with stronger EBHC competence. More specifically, educators who attended more than five education courses a year showed the strongest competence. A similar relationship has been reported in earlier studies and should motivate managers to allow educators numerous opportunities to update their competence, for example, participation in various education courses and research projects (Koskimäki et al., 2021, 2022; Kuivila et al., 2020). As such, the strong link between continuous education and EBHC competence cannot be overlooked.

The presented research revealed that social and healthcare educators in Finland share satisfactory levels of competence in the JBI model of EBHC, with educators working at universities and universities of applied sciences showing the highest levels of competence. Moreover, this subset of educators also participates more in continuous education, which is pivotal to strengthening their competence. Nevertheless, there were certain identified weaknesses, such as evidence synthesis and transfer, which suggests that these areas of EBHC should be emphasized during education. Educators are responsible for teaching future professionals, so their work to promote the JBI model of EBHC will play a significant role in the future quality of healthcare. Effective, collegial support in evidence implementation can also support the development of educators' EBHC competence (Immonen et al., 2022). Educators who work hard to master the different components of JBI model of EBHC will be better suited to teach evidence‐based approaches, which will be critical to maintaining a health professional identity that is aligned with current patient demands.

### 6.1 Limitations

The study included several limitations that should be considered when reviewing and applying the presented results. First, the participating educators rated their EBHC competence through self‐assessment, and this may have affected the results as it is plausible that certain respondents overestimated their level of competence. The response rate in the present study was low (12%) and may have impacted the quality of the presented results. However, the survey included all Finnish universities, all central universities of applied sciences and ten vocational colleges, which should provide a strong, nationwide perspective on the studied phenomenon. The results support previous studies on Finnish educators' generic evidence‐based healthcare competence and can be generalized at national level. Generalizability of the findings more broadly requires comparative studies because education of social and healthcare educators varies in different countries and can influence their EBHC competence.

The response rate may have been influenced by the choice of using an electronic survey, which may have been ignored by certain educators due to heavy workloads and the fact that they are exposed to various surveys, which is especially relevant to educators at vocational colleges. The questionnaire was designed in a way that it would be as easy as possible for participants to answer, that is, the survey included a succinct set of questions and respondents were provided with a limited number of ready‐made response options. Various methodological strategies which support effective and high‐quality data collection were employed.

## 7 CONCLUSIONS

The educators' EBHC competence level was mostly satisfactory or excellent. The development needs of competence were in the field of evidence synthesis and transfer. The educators' competence should be improved in evidence synthesis (systematic reviews, national care guidelines) as well as how to guide students' use of evidence in decision‐making with patient. The educators also should strengthen their role in transferring evidence into teaching. This could be enhanced through participation in various EBHC training courses, thematic conferences and mentoring projects.

The four distinct EBHC competence profiles were identified. The profiles help at identifying those educators who need the EBHC competence development the most. As the profiles revealed educators with lower competence, the improvement of EBHC competence could be included in regular development discussions. This would allow the educator to identify his/her own competence and related gaps, giving the supervisor a clear idea of the level of competence. Where appropriate, this could be used in leadership of competence development and to support educators' individual EBHC competence needs.

The results indicate that higher educational level can strengthen an educator's EBHC competence. Thus, more insight into EBHC competence among vocational college educators is needed to identify deeply the areas for competence development. They should be encouraged to participate in research and development projects. Teaching EBHC strengthens educators' competence and helps them teach EBHC holistically for future health professionals. Also, an integration of EBHC into the curricula could strengthen educators' competencies regardless of the level of the educational organization. Making EBHC visible in the curriculum will help educators familiarize themselves with EBHC in a planned way through the teaching content.

## AUTHOR CONTRIBUTIONS

Made substantial contributions to conception and design, or acquisition of data, or analysis and interpretation of data; KI, AMT, KM, MK. Involved in drafting the manuscript or revising it critically for important intellectual content; KI, AMT, KM, AO, SY, HP, OM, MK. Given final approval of the version to be published. Each author should have participated sufficiently in the work to take public responsibility for appropriate portions of the content; AMT, MK. Agreed to be accountable for all aspects of the work in ensuring that questions related to the accuracy or integrity of any part of the work are appropriately investigated and resolved. KI, AMT, KM, AO, SY, HP, OM, MK.

## FUNDING INFORMATION

The presented research did not receive any external funding.

## CONFLICT OF INTEREST STATEMENT

The authors can confirm that there are no conflicts of interest.

### PEER REVIEW

The peer review history for this article is available at https://www.webofscience.com/api/gateway/wos/peer‐review/10.1111/jan.16230.

## Data Availability

The data that supports the findings of this study are available in the supplementary material of this article.
